# Early decreased neutrophil responsiveness is related to late onset sepsis in multitrauma patients: An international cohort study

**DOI:** 10.1371/journal.pone.0180145

**Published:** 2017-06-30

**Authors:** Kathelijne M. Groeneveld, Leo Koenderman, Brian L. Warren, Saskia Jol, Luke P. H. Leenen, Falco Hietbrink

**Affiliations:** 1 Department of Surgery, UMC Utrecht, Utrecht, The Netherlands; 2 Department of Respiratory Medicine and Laboratory of Translational Immunolgy, UMC Utrecht, Utrecht, The Netherlands; 3 Division of Surgery, Tygerberg Hospital / Stellenbosch University, Cape Town, South Africa; University of Florida, UNITED STATES

## Abstract

**Background:**

Severe trauma can lead to the development of infectious complications after several days, such as sepsis. Early identification of patients at risk will aid anticipating these complications. The aim of this study was to test the relation between the acute (<24 hours) inflammatory response after injury measured by neutrophil responsiveness and the late (>5 days) development of septic complications and validate this in different trauma populations.

**Methods and findings:**

Two prospective, observational, cohort series in the Netherlands and South Africa, consisting of severely injured trauma patients. Neutrophil responsiveness by fMLF-induced active FcγRII was measured in whole blood flowcytometry, as read out for the systemic immune response within hours after trauma. Sepsis was scored daily. Ten of the 36 included Dutch patients developed septic shock. In patients with septic shock, neutrophils showed a lower expression of fMLF-induced active FcγRII immediately after trauma when compared to patients without septic shock (P = 0.001). In South Africa 11 of 73 included patients developed septic shock. Again neutrophils showed lower expression of fMLF induced active FcγRII (P = 0.001). In the combined cohort, all patients who developed septic shock demonstrated a decreased neutrophil responsiveness.

**Conclusions:**

Low responsiveness of neutrophils for the innate stimulus fMLF immediately after trauma preceded the development of septic shock during admission by almost a week and did not depend on a geographical/racial background, hospital protocols and health care facilities. Decreased neutrophil responsiveness appears to be a prerequisite for septic shock after trauma. This might enable anticipation of this severe complication in trauma patients.

## Introduction

Trauma is one of the major causes of morbidity and mortality worldwide, especially in young individuals [1]. Although the mortality rates of these patients have declined over the last decade, both mortality and morbidity rates remain high throughout the world [2, 3]. In addition, the World Health Organisation expects a 40% increase of deaths due to trauma between 2002 and 2030 due to fatalities associated with economic growth in low and middle income countries. Morbidity and mortality can be the direct result of injuries or indirectly due to post-injury complications such as organ dysfunction. Organ failure caused by septic shock is the most common cause of late (>5 days) death. In some cases the cause is clear, such as a bowel perforation, on the other hand, these inflammatory complications can occur without obvious cause and are thought to be the result of a non-functioning systemic immune response in which polymorphonuclear neutrophils (PMNs) play a pivotal role [4–6].

In previous studies we have investigated the functional phenotype of neutrophils in trauma patients [7, 8]. We used the responsiveness (fMLF induced active FcγRII) of neutrophils in the peripheral blood to gain insight in the inflammatory status of the host [9, 10]. Reduced responsiveness of neutrophils towards fMLF was related to trauma severity and lasted a week [10]. Recent observations of a neutrophil life span of 5.4 days in peripheral blood and a post-mitotic transfer time of 6–7 days in the bone marrow support the hypothesis that the neutrophil compartment can be aberrantly regulated in trauma for up to 5 days, with no renewal of cells from the mitotic pool [11]. The aim of the current project was to investigate whether a relation exists between the extend of the initial (day of admission) immunological response after injury and the late (>5 days) development of inflammatory complications (e.g. septic shock). The responsiveness of neutrophils was used as marker. The method of analysis was validated in two cohorts on two continents and in two completely different health care systems, different racial background and different trauma mechanisms to determine the feasibility as prognostic marker.

## Materials and methods

### Patient inclusion UMC Utrecht

A consecutive series of severely injured adult (≥ 18 years) patients, with an Injury Severity Score (ISS) >16, who required intensive care support (ICU) in the University Medical Centre Utrecht (UMCU) was included (for demographical data see Table 1). Their expected ICU stay was ≥ 3 days. Patients receiving any therapy reducing host defence (e.g. immunosuppression, chemotherapy, or long term steroid use) or with a pre-existing condition that alters neutrophil function (e.g. malignant lymphoma or leukaemia) were excluded from the study. Elderly patients (>80 years) were also excluded to minimize the effect of age related changes in the efficiency of the immune system [12].

**Table 1:** Patient demographics.

	UMCU	TBH
Number of patients	36	73
Male/Female (N)	30/6	57/16
Age (years)	45 (20.1)	31 (8.7)
ISS	24 (10.9)	27 (8.7)
APACHE II Score	14 (7.5)	11 (7.1)
Time on ICU (days)	16 (12.7)	8 (10.7)
Time on ventilation (days)	15 (12.7)	5 (5.4)
Overall mortality	2	11
Nature of trauma (N)	Blunt = 94%	Blunt = 61%
*MVA*	24	37
*Burns*	0	10
*Other blunt injury*	10	7
*Penetrating (e*.*g*. *GSW)*	2	19
Neurotrauma	20	27
Septic shock (N)	10	11
Mortality due to MODS (N)	1	3

Numbers are depicted as mean (±SD), unless otherwise specified

Listed are the characteristics of the trauma patients who were admitted.

UMCU = University Medical Centre Utrecht; TBH = Tygerberg Hospital; N = number of patients, ISS = Injury Severity Score; = intensive care unit; MVA = motor-verhicle accident; GSW = gunshot wound; Neurotrauma = abbreviated injury scale head (AIS Head) >3. MODS = Multiple Organ Dysfunction Syndrome

The attending physician was responsible for the treatment plan and other decisions concerning the patients. The flowcytometry results of the patients were compared with results from ten age- and sex matched healthy controls. The institutional ethical committee approved the study and written consent was obtained from all patients or their legal representatives in accordance to the protocol: “Medisch Ethische Toetsingscommissie van het UMC Utrecht” (S2 File). Patient demographics have been previously published for the kinetics of the pathophysiological process [10].

### Patient inclusion Tygerberg Hospital

To validate the findings and minimise the effect of geographic, racial, trauma mechanism, economic and healthcare associated influences on the initial results, a second series of patients was included at the Trauma Unit of the Tygerberg Hospital (TBH) in Cape Town of the Stellenbosch University in South Africa. Tygerberg Hospital is a tertiary hospital with a level 1 Trauma Unit. It houses the Health Science Faculty of the University of Stellenbosch and a National Training Centre and Research Unit with high end equipment and modern infrastructure, including laboratory facilities. At the Trauma Unit a consecutive series of adult (≥ 18 years) patients with multiple injuries (ISS ≥ 16) was included. Patients who died within three hours of admission were excluded.

As in the Netherlands, patients receiving any therapy reducing host defence, with a pre-existing condition that alters neutrophil function or aged >80 years were excluded from the study. Calculations of Injury Severity Scores, documentation of systemic inflammatory complications and sampling and analysis of blood samples were performed as described above. The flowcytometry results of the patients in the Tygerberg Hospital were compared with results from twenty South-African age and sex matched healthy controls. The local institutional ethical committee approved the study and written consent was obtained from all patients or their legal representatives in accordance to the protocol: “Committee for Human Research Tygerberg” (S1 File).

### Clinical parameters

On admission the Injury Severity Score (ISS) and APACHE II score were calculated[13, 14]. During admission the presence of late systemic inflammatory complications (e.g. sepsis/septic shock) was recorded on a daily basis according to the clinical criteria [15, 16]: Based on the study protocol, sepsis was documented as two or more of the SIRS criteria, with documented or suspected infection. Patients were diagnosed with a septic shock when they were in a state of severe sepsis with persistent hypotension despite adequate fluid resuscitation, in the absence of other causes for hypotension. While the study was in progress, new sepsis definition were published [17]. To comply with the new sepsis definitions, data were recalculated to identify patients with “septic shock”, while the remaining patients were defined as “no-septic shock” [17].

### Sampling and analysis

A single blood sample was taken on arrival or when secondary survey and initial surgical procedures were completed (<24 hours). Blood was sampled before any surgical procedures were performed and at the latest twelve hours after the injuries were sustained. The blood (6 mL) was collected in a Vacutainer® with sodium heparin as anticoagulant, immediately cooled on melting ice and kept on ice during the whole procedure. Within two hours of sampling, blood samples were analysed for neutrophil phenotype and responsiveness with the use of flowcytometry. The analysis of neutrophil receptor expression profiles was described previously [8]. The following monoclonal antibodies were purchased commercially: FITC-labeled IgG1 (clone DD7) from Chemicon, Hampshire, United Kingdom, FITC-labeled IgG2 (clone MRC OX-34), from Celtic Molecular Diagnostics, Serotec, Mowbray, RPE-labeled IgG1 (clone MRC OX-34), AbD Serotec, Oxford, United Kingdom, RPE-labeled IgG2a (clone MRC OX-34), Alexa-labeled IgG1 (clone MOPC-21) from Scientific Group, BD Pharmingen, Milnerton and RPE-labeled CD11b (clone 2LPM19c) from Dako, Glostrup, Denmark. A monoclonal phage antibody, which recognizes active FcγRII (active CD32), was manufactured in the Department of Respiratory Medicine at the University Medical Centre Utrecht (MoPhap A27). Interleukin-6 (IL-6) analysis was performed using a sandwich ELISA (Pierce Biotechnology Inc. IL, United States) as described by the manufacturer.

The monoclonal antibodies were added 1:20 to whole blood and incubated for 60 minutes on ice. After incubation, the red cells were lysed with ice-cold isotonic NH4Cl. After a final wash with PBS2+ (phosphate buffered saline with added sodium citrate (0.38% wt/vol) and pasteurized plasma proteins (10%vol/vol), the cells were analyzed by flowcytometry (FACScalibur Flowcytometer, Becton&Dickinson, Mountain View, CA). Neutrophils were identified according to their specific forward- and sideward-scatter signals.

Neutrophil responsiveness was determined by expression of activation marker CD11b, which recognized the αM part of the MAC-1 complex (CD11b/CD18), a read-out for neutrophil activation, because this receptor is up-regulated during fusion of secretory vesicles and specific granules [18, 19]. Inside-out activation of active FcγRII is a sensitive read-out for in situ activation of neutrophils [20]. To evaluate the responsiveness of the circulating neutrophils for bacterial derived activating agonists, the expression of active FcγRII was also measured after 5 minutes of whole blood stimulations with formyl- methionyl-leucyl-phenylalanine (fMLF 10^-6^M) at 37°C in vitro.

To standardize analysis, flowcytometer settings were synchronized between the Dutch and South African flowcytometers. In addition, the read-out was maximized to 10000 AU, which could be analysed in healthy controls.

### Statistics

Statistical analysis was performed using IBM SPSS version 24. Data from individual experiments are depicted as mean fluorescence intensity (MFI) in arbitrary units (AU) of at least 10.000 events. Statistical analysis was performed with the students T-test, to compare healthy controls with the trauma patients. When comparing more than two groups ANOVA was used. In the case of laboratory parameters, non-parametric tests were used as these data were not normally distributed (Mann Whitney U test and Kruskall Wallis H test). Statistical significance was defined as p<0.05. ROC analysis was performed for the functional phenotype. Optimal cut-off point was determined at a sensitivity of 0.90. The outcome of the first cohort was verified in the second cohort using the optimal cut-off point.

## Results

### Demographics

#### University Medical Center Utrecht (the Netherlands)

In total, forty patients fulfilled the inclusion criteria. Two patients were transferred to another hospital and in two patients informed consent was not obtained, resulting in 36 patients for analysis consisting mainly of Caucasian males. Of these patients, 10 patients developed septic shock. Two patients with a late phase septic shock died due to multiple organ dysfunction syndrome (Table 1). Nine of the 10 patients who developed septic shock, fulfilled the criteria later than 5 days after admission. One patient suffered bowel injury and developed septic shock on the third day of admission.

#### Tygerberg Hospital Cape Town (South Africa)

Seventy-six patients met the inclusion criteria. One patient was transferred to another hospital and two patients had a pre-existing condition that altered their immune system. Thus 73 patients remained for analysis, consisting mainly of black African males. Of these 11 patients developed septic shock. Eleven patients died during their admission. Cause of death for 3 patients was multiple organ dysfunction syndrome during a late phase septic shock. Seven other patients died within 6 hours to nine days, because of complications directly related to their initial injuries. In one patient the cause of death is unknown (Table 1). The patients who developed sepsis and septic shock, fulfilled the criteria between 3–11 days after admission.

### Injury and disease severity on admission

All patients were severely injured (Table 1). No statistically significant differences were found in the score for injury severity at admission between patients who developed septic shock and patients without inflammatory complications (Table 2). Although differences were small, larger number of patients might result in significant differences. There was also no significant difference in leukocyte numbers (results not depicted). Compared to healthy individuals, plasma IL-6 levels were increased in all patients on admission. However, values differed considerably due to large interpersonal variation and failed to reach statistical significance (UMCU P = 0.497 and TBH P = 0.606 for no-septic shock vs septic shock).

**Table 2:** Injury severity and complications.

	Mean (SD)	No complication	Septic shock	P-value
UMC	ISS	23 (9.7)	29 (12.7)	0.191
	APACHE II	13 (7.2)	18 (7.2)	0.083
TBH	ISS	27 (9.1)	27 (6.0)	0.881
	APACHE II	10 (7.3)	12 (5.2)	0.296

UMCU = University Medical Centre Utrecht; TBH = Tygerberg Hospital; ISS = Injury Severity Score; ICU = inte**n**sive care unit.

### Initial inflammatory response

#### University Medical Center Utrecht (the Netherlands)

**Fig 1:**
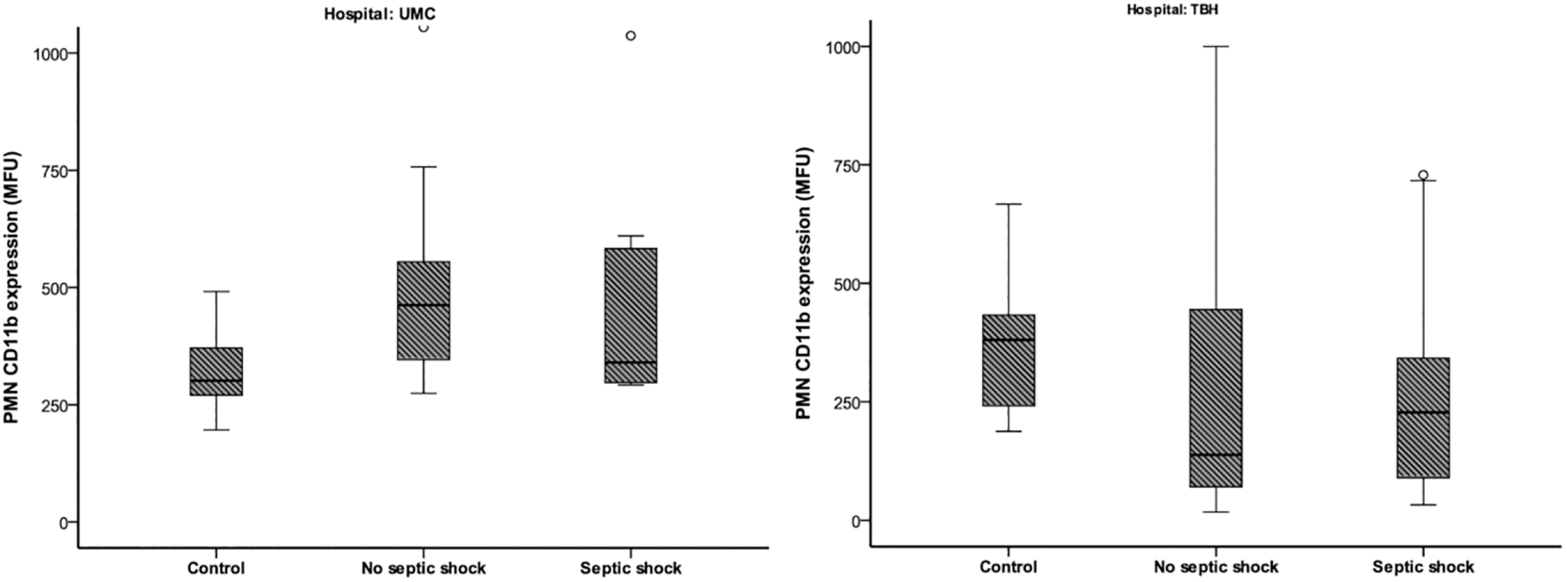
CD11b expression on PMNs. Relation between CD11b expression on PMNs (mean fluorescence intensities; MFI in arbitrary units; AU) and the development of inflammatory complications. Complications are depicted according to the sepsis consensus conference and divided by hospital: University Medical Center Utrecht (UMC), Tyberberg Hospital Stellenbosch (THB). In UMC (**A**) a significant increase was found in all patients (p<0.006) compared to controls. No differences were found between patients. In TBH (**B**) no differences were found between patients and controls, nor between patients with septic shock and all other patients.

**Fig 2:**
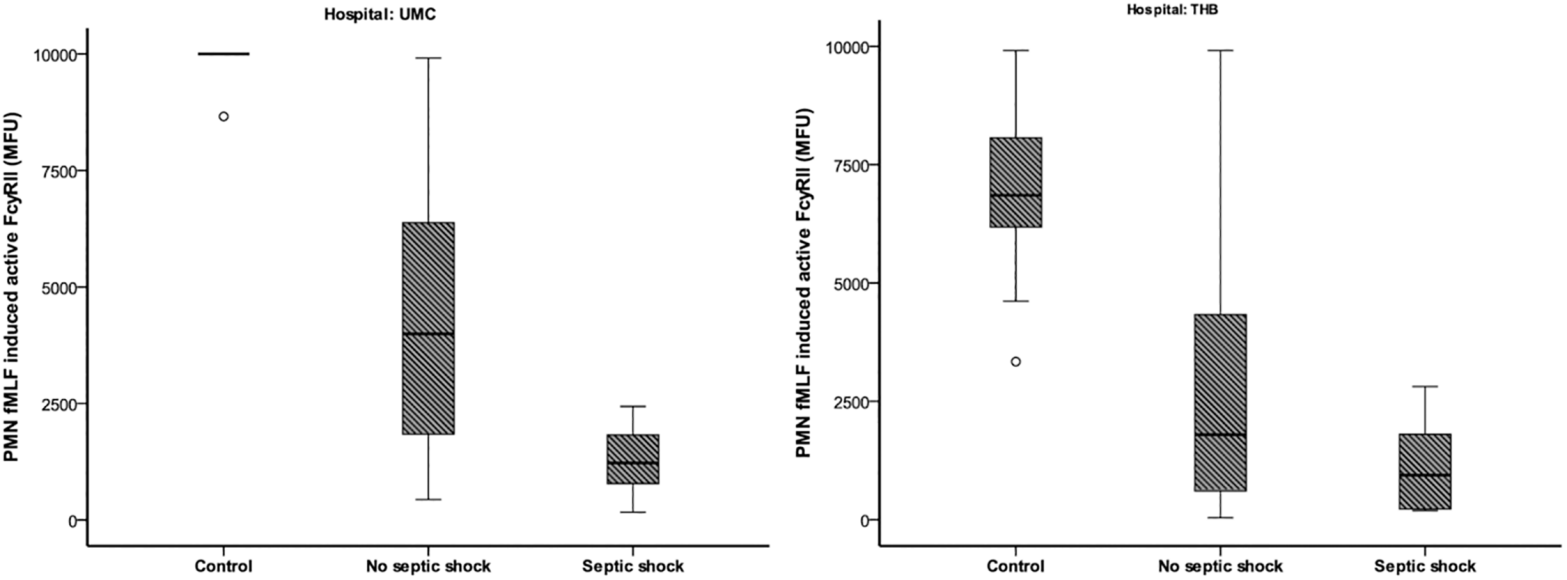
FMLF-induced FcγRII expression on PMNs. Relation between fMLF-induced FcγRII expression on PMNs (mean fluorescence intensities; MFI in arbitrary units; AU) and the development of inflammatory complications. Complications are depicted according to the sepsis consensus conference and divided by hospital: University Medical Center Utrecht (UMC), Tyberberg Hospital Stellenbosch (THB). In UMC (**A**) a significant decreased responsiveness was found in all patients (p<0.001) compared to controls. In addition, a statistically significant difference was found between patients with septic shock and patients without (p<0.001). In TBH (**B**) a significant decreased responsiveness was found in all patients (p<0.001) compared to controls. A statistically significant difference was found between patients with and without septic shock (p = 0.001).

The expression of CD11b was statistically significant increased compared to controls (P = 0.006), but no relation with septic shock was found (Fig 1A). The responsiveness of neutrophils after ex vivo fMLF stimulation in inducing inside out control of active FcγRII was significantly decreased in patients compared to controls (P<0.001) and was related to patients who developed septic shock compared to those who did not (P<0.001) (Fig 2A). The outcome values for the old sepsis criteria were provided in S1 Fig.

#### Tygerberg Hospital Cape Town (South Africa)

Immediately after trauma the expression of CD11b was not statistically significant different between patients and controls. No clear relation with septic shock was found (Fig 1B). As in patients in the Dutch cohort, the responsiveness of neutrophils towards fMLF for active FcγRII was significantly decreased (P<0.001) and the amplitude related to patients who developed septic shock and those who did not (P<0.001, Fig 2B).

### Validation of the functional neutrophil phenotype as possible prognostic marker

No differences were found between different types of trauma (blunt, penetrating or burns; with or without neurotrauma), concerning fMLF induced active FcγRII. As the pattern of neutrophil function was similar, all trauma mechanisms were analysed together. A ROC was plotted for fMLF induced active FcγRII on neutrophils in relation to the development of septic shock for admitted patients and the areas under the curve (AUC) were calculated (UMCU = 0.805 and TBH = 0.747, combined = 0.706). A combined sensitivity of 90% was achieved at approximately 2500 AU. However, specificity was low (20%), thus this parameter probably has mainly a negative predictive value. No patients who demonstrated fMLF induced expression of active FcγRII on neutrophils of >3000 AU developed septic shock in either cohort.

## Discussion

In this international study we analysed the inflammatory response that follows multiple injuries in the first day. The initial decreased responsiveness of circulating neutrophils (fMLF-induced active FcγRII) was related to the development of late onset septic complications after >5 days. All patients who developed septic shock demonstrated a severe decreased neutrophil responsiveness, indicating this reduced responsiveness is a prerequisite.

The development of inflammatory complications such as septic shock is based on many factors. An entry point for bacteria, colonization status and duration of exposure are part of this multifactorial mechanism leading to septic shock [21]. However, all patients with a responsive phenotype of circulating PMNs towards fMLF in the initial phase had an uneventful course, which indicates that a nonfunctioning immune response might be a prerequisite in such a model. Although the fMLF-induced active FcγRII might not be sufficient to solely predict the occurrence of septic shock, it appears to be a sensitive marker and depicts an essential part of the immune system involved in the pathophysiology of insult by injury and the development of sepsis and septic shock.

It is tempting to speculate on the mechanism that can explain this relation between the initial refractory phenotype of neutrophils and late development of septic shock. New insights into neutrophil biology can shed some light on this putative mechanism. Recent studies are consistent with a relatively long lifespan of neutrophils of around 5 days and a postmitotic transfer time between 6–7 days [11, 22–24]. Normally, the immune system is characterized by normal functioning neutrophils, but it is tempting to speculate that the massive release of neutrophils from the bone marrow after trauma depletes the storages in the bone marrow. This is supported by large amounts of banded cells and sometimes even immature cells (i.e. metamyelocytes). In addition, the released neutrophils will become aberrantly regulated because of the ‘storm’ of DAMPs and PAMPs. As the system is inherently slow (it takes days to make new neutrophils in the bone marrow) the deregulated neutrophils will stay present for multiple days. The more the cells are activated the more pronounced the deregulation of the neutrophil compartment will be [9]. Particularly, between 5 days (peripheral life span) and 7 days (time to produce new neutrophils from myelocytes) the situation might support aberrant innate immune control, that makes the patient at risk for infectious complications such as septic shock [23–25]. Early identification of patients at high risk of septic shock and identification of the underlying pathology would greatly aid in the identification of patients at risk and adaptation of the treatment regime [7, 26, 27].

Experimental studies strongly suggest that a direct relation is present between trauma severity, subsequent tissue damage and neutrophil dysfunction. In addition, leukocyte phenotype could assist in diagnosing sepsis [21, 28]. In previous studies consisting of a more homogeneous trauma population, a relation in some degree could be demonstrated between the extent of trauma expressed by clinical scores and the magnitude of decreased responsiveness of neutrophils characterized by a decreased fMLF-induced active FcγRII[8, 9]. In the present study consisting of heterogeneous trauma populations, we could confirm this finding by showing that a more profound initial systemic (cellular) innate immune response directly after trauma was found in those patients ultimately developing septic shock. Since the impaired responsiveness of circulating neutrophils for fMLF already occurred within 24 hours after trauma, the induction of this refractoriness clearly preceded clinical symptoms by almost a week! In addition to the above mentioned relation, this study shows that these results do not depend on differences in patients’ population, trauma mechanism, researcher, flowcytometry equipment and analysis settings, treatment protocols in the Emergency Department and Health Care facilities, since we demonstrated a similar phenomenon in both studies/institutions. The analysis were performed on whole blood, which could possibly have attributed to the stability of these results [29, 30].

The patients included in this study developed a relatively high number of complications and had a high mortality rate. This was partly due to the inclusion criteria and hospital settings, especially in the UMCU[10]. Because of labour intensive analysis, inclusion criteria were set to have a high possibility of patients developing sepsis. In short, the most critically ill patients who survived the first 72 hours but remained on the ICU were selected and therefore more prone for the development of septic complications. During the inclusion period, all trauma patients who developed septic shock were included. Furthermore, in the South African population, a substantial number of patients had burns and/or penetrating injuries, whereas in the Dutch population all patients suffered blunt injuries. Penetrating injuries are generally underrepresented in a western trauma population. It has been suggested that within the same Injury Severity Score, penetrating trauma leads to a less pronounced inflammatory response and has a lower mortality rate compared to blunt trauma [31]. However, in the present study, no differences between trauma mechanism and immune response could be demonstrated. To further stretch the differences between hospitals, in TBH, a broader variety in days was found between admission and development of sepsis or septic shock. In addition, in TBH seven patients died due to their injuries, before they could develop an inflammatory complication.

Despite all differences between our two study cohorts, in both groups the decreased responsiveness of neutrophil active FcγRII towards fMLF seemed to be a prerequisite for the development of inflammatory complications such as septic shock. This initial essential step in the pathophysiology after trauma preceded septic shock by almost a week.

Distinct multiple cellular phenotypes were not present at a certain time in the peripheral blood, as most patients demonstrated quite narrow staining curves. Multiple staining per cell was not performed, therefore multidimensional analysis of subtypes could not be performed. Based on single staining, a relative homogenous population of aberrant neutrophils are present in the blood after trauma. Larger cohort studies should be performed to allow for multivariate analysis. Our study supports the development of an automated multicolour flowcytometric analysis of responsiveness of blood neutrophils for the analysis of early dysfunction of the innate immune response in trauma patients.

## Ethics statement

The local institutional ethical committees approved the study and written consent was obtained from all patients or their legal representatives in accordance to the protocol on both locations: “Medisch Ethische Toetsingscommissie van het UMC Utrecht” and “Committee for Human Research Tygerberg”.

## Supporting information

S1 Dataset Additional source data. (DOCX)

S1 Fig Neutrophil parameters in relation to old sepsis definitions. Similar differences are seen for the old sepsis definitions between patient and control and between patients with and without septic shock, as compared with the new sepsis definitions.
(TIF)

S1 File N0804117_Approval EASY: Medical Ethical Committee approval letter TBH. (PDF)

S2 File 06-249-O_Approval NERTHUS: Medical Ethical Committee approval letter UMCU. (PDF)
