# Supplementary material for: Early decreased neutrophil responsiveness is related to late onset sepsis in multitrauma patients: An international cohort study
Source: PLoS One. 2017 Jun 30;12(6):e0180145. doi: 10.1371/journal.pone.0180145 (PMC5493351; doi:10.1371/journal.pone.0180145)
Supplement: S1 Dataset — (DOCX) [file pone.0180145.s001.docx]

**MAC-1** (mean fluorescence intensity)

| **UMC Utrecht** | **Mean** | **Median** | **95%-CI** |
| --- | --- | --- | --- |
| Control | 321 | 301 | 242 - 400 |
| No complications | 427 | 422 | 302 - 551 |
| SIRS | 464 | 502 | 328 - 600 |
| Sepsis | 536 | 459 | 384 - 688 |
| Septic shock | 471 | 340 | 281 - 660 |
|  |  |  |  |
| **Tygerberg Hospital** |  |  |  |
| Control | 365 | 380 | 300 - 431 |
| No complications | 209 | 109 | 82 - 335 |
| SIRS | 264 | 126 | 135 - 392 |
| Sepsis | 512 | 417 | 323 - 701 |
| Septic shock | 284 | 228 | 100 - 469 |

**fMLF induced acitve FcyRII** (mean fluorescence intensity)

| **UMC Utrecht** | **Mean** | **Median** | **95%-CI** |
| --- | --- | --- | --- |
| Control | 9832 | 10000 | 9436 - 10228 |
| No complications | 5172 | 4656 | 1279 - 9065 |
| SIRS | 4117 | 4091 | 2169 - 6065 |
| Sepsis | 3868 | 2919 | 1421 - 6315 |
| Septic shock | 1285 | 1225 | 668 - 1901 |
|  |  |  |  |
| **Tygerberg Hospital** |  |  |  |
| Control | 7038 | 6854 | 5947 – 8129 |
| No complications | 4792 | 5186 | 3126 – 6459 |
| SIRS | 2044 | 1946 | 1423 - 2666 |
| Sepsis | 1629 | 589 | 359 - 2900 |
| Septic shock | 1098 | 939 | 506 - 1690 |
