# Supplementary material for: Early decreased neutrophil responsiveness is related to late onset sepsis in multitrauma patients: An international cohort study
Source: PLoS One. 2017 Jun 30;12(6):e0180145. doi: 10.1371/journal.pone.0180145 (PMC5493351; doi:10.1371/journal.pone.0180145)
Supplement: S1 File — (PDF) [file pone.0180145.s003.pdf]

9 July 2008

Prof LPH Leenen
Dept of Surgery

RESEARCH PROJECT : "Early identification of late onset sepsis with netrophil phenotype in multi trauma patients: EASY- study."
PROJECT NUMBER : N08/04/117

At a meeting of the Committee for Human Research that was held on 7 May 2008 the above project was approved on condition that further information that was required, be submitted.

This information was supplied and the project was finally approved on 9 July 2008 **for a period of one year from this date**. This project is therefore now registered and you can proceed with the work.

Please quote the above-mentioned project number in ALL future correspondence.

Please note that a progress report (obtainable on the website of our Division: www.sun.ac.za/knowledgepartner/committees_CHR.htm) should be submitted to the Committee before the year has expired. The Committee will then consider the continuation of the project for a further year (if necessary). Annually a number of projects may be selected randomly and subjected to an external audit.
Translations of the consent document in the languages applicable to the study participants should be submitted.

Federal Wide Assurance Number: 00001372
Institutional Review Board (IRB) Number: IRB0005239

The Committee for Human Research complies with the SA National Health Act No.61 2003 as it pertains to health research and the United States Code of Federal Regulations Title 45 Part 46. This committee abides by the ethical norms and principles for research, established by the Declaration of Helsinki, the South African Medical Research Council Guidelines as well as the Guidelines for Ethical Research: Principles Structures and Processes 2004 (Department of Health).

Kind regards

Mertrude Davids
RESEARCH DEVELOPMENT AND SUPPORT (TYGERBERG)
Tel: +27 21 938 9207 / E-mail: mertrude@sun.ac.za

Approval Date: 9 July 2008 **Expiry Date: 9 July 2009**
