# Supplementary material for: Early decreased neutrophil responsiveness is related to late onset sepsis in multitrauma patients: An international cohort study
Source: PLoS One. 2017 Jun 30;12(6):e0180145. doi: 10.1371/journal.pone.0180145 (PMC5493351; doi:10.1371/journal.pone.0180145)
Supplement: S2 File — (PDF) [file pone.0180145.s004.pdf]

Divisie Heelkundige Specialismen
Afdeling Heelkunde
t.a.v. F. Hietbrink
Huispost : G04.228

Mw. M.A.C. van Groenestijn
Telefoon 030-2506376 (09.30-12.00uur)
Fax 030-2505400
Huispost D 01.343
E-mail metc@umcutrecht.nl
Info www.umcutrecht.nl/metc
of via Scoop, index, m, metc

Datum
12 januari 2007

Ons kenmerk
AvG/vb/07/00817

Onderwerp
METC-protocolnummer 06-249/O
Toestemming Raad van Bestuur

Geachte heer, mevrouw,

Hierbij geeft de Raad van Bestuur, gehoord hebbende het positieve oordeel in de zin van de WMO van de Medisch Ethische Toetsingscommissie van het UMC Utrecht d.d. 12 december 2006, toestemming voor de uitvoering van onderzoeksvoorstel met METC-protocolnummer 06/249, getiteld **"Paralysis of neutrophils in intensive care patients and the development of sepsis"**.

Ik wens u veel succes bij de uitvoering van uw onderzoek.

Met vriendelijke groeten,
namens de Raad van Bestuur,

Prof. dr. G.H. Blijham,
voorzitter

cc: METC

bijlage: positief oordeel brief
